# Retinoic Acid Signaling Is Compromised in DSS-Induced Dysbiosis

**DOI:** 10.3390/nu14142788

**Published:** 2022-07-06

**Authors:** Yongchun Li, Lili Sheng, Prasant Kumar Jena, Miranda Claire Gilbert, Yu-Jui Yvonne Wan, Hua Mao

**Affiliations:** 1 Department of Gastroenterology, Zhujiang Hospital, Southern Medical University, Guangzhou 510282, China; liyongchun@fssnhormyy2.wecom.work; 2 Department of Infectious Diseases, The Six Affiliated Hospital, South China University of Technology, Foshan 528200, China; 3 Department of Pathology and Laboratory Medicine, University of California Davis, Sacramento, CA 95817, USA; llsheng@shutcm.edu.cn (L.S.); prasantjenacps@gmail.com (P.K.J.); miranda.claire3@gmail.com (M.C.G.)

**Keywords:** vitamin A, nuclear receptor, gut microbiota, obesity, malnutrition

## Abstract

Obesity and malnutrition both cause dysbiosis and dampen retinoic acid (RA) signaling pathways, which play pivotal roles in biological processes. The current study evaluates a hypothesis that colitis-associated dysbiosis also has systemic negative impacts on RA signaling. Thus, we studied the effects of inflammation, under a vitamin A-sufficient condition, on RA signaling using mouse colitis models induced by dextran sulfate sodium. That data showed that intestinal inflammation resulted in reduced RA signaling in the liver, brain, gut, and adipose tissues measured by analyzing the expression of genes encoding for the synthesis, oxidation, transport, and receptor of RA. The expression of RA-regulated gut homing molecules including α4β7 integrin, and CCR9, along with MADCAM1 were all reduced in colitis mice revealing compromised immunity due to reduced RA signaling. The data also showed that the development of colitis was accompanied by dysbiosis featured with reduced Lactobacillaceae and Verrucomicrobiaceae but an expansion of Erysipelotrichaceae and others. Colitis resulted in reduced butyrate-producing bacteria and increased methane-generating bacteria. Additionally, dysbiosis was associated with induced Il-1β, Ifn-γ, and Tnf-α mRNA but reduced Il-22, Il-17f, and Rorγt transcripts in the colon. Together, intestinal inflammation inhibits RA signaling in multiple organs. RA is essential in regulating various biological processes, it is critical to detect RA signaling reduction in tissues even when vitamin A deficiency is absent. Moreover, probiotics can potentially prevent dysbiosis and reverse compromised RA signaling, having systemic health benefits.

## 1. Introduction

Obesity and malnutrition are risks for inflammation. The prevalence of malnutrition in inflammatory bowel disease (IBD) patients can be as high as 85%. Malnutrition causes vitamin A deficiency. Similarly, overfeeding causes compromised retinoic acid (RA) signaling. RA, the biologically active metabolite of vitamin A, has organ-specific biological functions. In the liver, RA regulates metabolism, differentiation, and regeneration. In the brain, RA maintains neuroplasticity. In adipose tissue, RA induces adipose tissue browning. RA also controls the gut homing of immune cells and has a critical role in regulating immunity. Thus, RA signaling deficiency has a serious systemic impact.

The gut and liver are anatomically linked. The hepatic portal vein carries blood drained from the gastrointestinal tract and spleen to provide about 75% of the liver’s blood supply. Thus, the liver is constantly exposed to intestine-derived products such as cytokines and metabolites. Therefore, inflamed intestines have an impact on liver health.

IBD is a chronic and relapsing inflammatory disorder of the gastrointestinal tract. The etiology is not fully understood and dysbiosis is one of the characteristics. The prevalence of IBD has increased worldwide in parallel with the incidence of obesity, stressing the significance of diet and dysbiosis in IBD development [1,2,3]. The current study examines the impact of colitis on RA signaling as well as microbiota structure.

Microbiota play a pivotal role in control of host immunity [4,5]. One mechanism by which gut microbes regulate host immunity is via bacteria-generated metabolites, of which short-chain fatty acids have been extensively studied. Short-chain fatty acids are generated by the bacterial fermentation of fibers. Among them, propionic acid, butyric acid, and valeric acid possess histone deacetylation properties and thereby can modify host gene expression via epigenetic mechanisms [6,7].

It is important to note that RA and bile acids share redundant effects such as regulating lipid metabolism and insulin sensitivity in part because their receptors, retinoid x receptor (RXR) and farnesoid X receptor (FXR), form heterodimers, which jointly regulate transcription [8]. Additionally, RA regulates bile acid homeostasis. In mice, RA reduces serum cholesterol, triglyceride, and bile acid levels [9]. RA also induces white adipose tissue browning by increasing beige adipogenesis and adipose vascularity [10]. DNA binding data revealed extensive crosstalk among RARα, pregnane X receptor (PXR), liver X receptor (LXR), FXR, and peroxisome proliferator-activated receptor α (PPARα) in regulating RA/RXRα-dependent gene expression in the liver [9,11]. Thus, RA signaling has a significant health impact.

Although vitamin A deficiency is uncommon in developed Western countries, it is alarming that obesity causes reduced RA signaling [12]. Furthermore, long-term Western diet intake, which induces systemic inflammation, also reduces RA signaling in microglia [13]. Moreover, in the brain, vitamin A deficiency causes amyloid deposition in the liver [14]. Compromised RA signaling also leads to neurodegeneration and the advancement of Alzheimer’s disease [13,15]. In contrast, RA supplementation, which simultaneously boosts RA signaling in the liver, can reduce inflammatory cytokines in microglia and astrocytes, which are activated in Alzheimer’s disease [16]. It would be important to know whether colitis affects RA signaling in the brain.

RA has a central role in orchestrating intestinal immunity [17]. In the gut, RA functions as an immunomodulator by inducing gut-homing molecules α4β7 integrin and C-C chemokine receptor 9 (CCR9) in T and B cells [18,19]. Moreover, RA signaling plays a crucial role in maintaining the stability of CD4+ T cell lineages, which are essential for immune homeostasis [20]. RA expands Foxp3(+) regulatory T cells and suppresses the differentiation of naive T cells into T helper type (Th) 17 cells leading to a tolerogenic environment [21]. However, RA is also indispensable for proper Th1, Th2, and Th17 responses. Thus, it is of interest to understand whether colitis has an impact on intestinal RA signaling.

Although previous studies indicate that RA can improve colitis in human and animal models [22,23], the role of RA is not straightforward, and is sometimes even contradictory [24,25]. A lack of RA leads to impaired immunity, whereas excess RA can potentially promote inflammation [26]. It would be essential to uncover ways to boost RA signaling.

Together, diet-induced dysbiosis affects RA signaling and it would be interesting to study whether chemical-induced dysbiosis has the same negative effect. Thus, we studied the impact of inflammation, under a vitamin A-sufficient condition, on RA signaling. This study provides evidence that colitis-associated dysbiosis affects RA signaling in the liver, brain, adipose tissues, and hints at the potential therapeutic application of using diet or probiotics to prevent colitis or RA signaling reduction.

## 2. Materials and Methods

### 2.1. Mouse Colitis Model

Specific pathogen-free C57BL/6 male mice (Jackson Laboratory, Sacramento, CA, USA) aged eight weeks old were housed in steel micro isolator cages at 22 °C with a 12 h light/dark cycle. Mice were fed with a chow diet (24% protein, 58% carbohydrate, 18% fat, Teklad Global 18% Protein Rodent Diet; Harlan Teklad diet, Placentia, CA, USA). Mice were randomly assigned to the control or DSS group (n = 9 per group). DSS was provided in drinking water (5%, 36–50 kDa, MP biosciences, Heidelberg, Germany) for 7 days [27].

Pathological evaluation of the ileum and colon was performed and scoring criteria are detailed in Table 1 and Table 2 [28,29]. Animal experiments were conducted in accordance with the NIH Guidelines for the Care and Use of Laboratory Animals under protocols approved by the Institutional Animal Care and Use Committee of the University [30].

### 2.2. Gene Expression

RNA was isolated using TRIzol (Invitrogen, Carlsbad, CA, USA) and reverse transcribed into cDNA. Real-time quantitative polymerase chain reaction (RT-qPCR) was performed on an ABI 7900HT Fast real-time PCR system using Power SYBR Green PCR Master Mix (Applied Biosystems, Foster City, CA, USA). The mRNA levels were normalized to the Ct values of Gapdh mRNA. The primers used for qPCR are shown in Table 3.

### 2.3. Quantification of Bacterial DNA

Cecum content DNA was purified using the ZR Fecal DNA miniprep kit (Zymo Research, Irvine, CA, USA). DNA was quantified by NanoDrop 8000 (Thermo Fisher Scientific, Wilmington, DE, USA), and amplified using primers based on published sequences [29,31]. Primers used for the qPCR of bacteria gene copy number are shown in Table 4. A dissociation step was included to analyze the melting profile of amplified products. qPCR was also carried out using 10-fold serial diluted synthetic DNA fragments of a bacterial gene with known concentrations. Bacterial DNA concentration was calculated using standard curves of diluted synthetic DNA fragments.

### 2.4. 16S rRNA Gene Pyrosequencing

Illumina sequencing of barcoded 16S rRNA gene amplicons of genomic DNA was carried out based on published methods [32]. The variable region 4 of the 16S rRNA gene was amplified and sequenced. DNA sequence reads were demultiplexed and classified with the use of custom python-based dbcAmplicons to identify and assign the reads by both expected barcode and primer sequences [32]. The Ribosomal Database Project Bayesian classifier was used to assign sequences to phylotypes [33]. Reads were assigned to the first Ribosomal Database Project taxonomic level with a bootstrap score ≥ 50.

### 2.5. Statistical Analysis

Unpaired Student’s *t*-test was performed by using GraphPad Prism software version 6.0 (GraphPad Software, Inc., La Jolla, CA, USA). Data were expressed as mean ± SD. Differences between groups in microbiota phylum and family level were calculated using the Mann–Whitney U-test. *p* < 0.05 was considered statistically significant.

## 3. Results

### 3.1. Mouse Colitis Model

**Figure 1:**
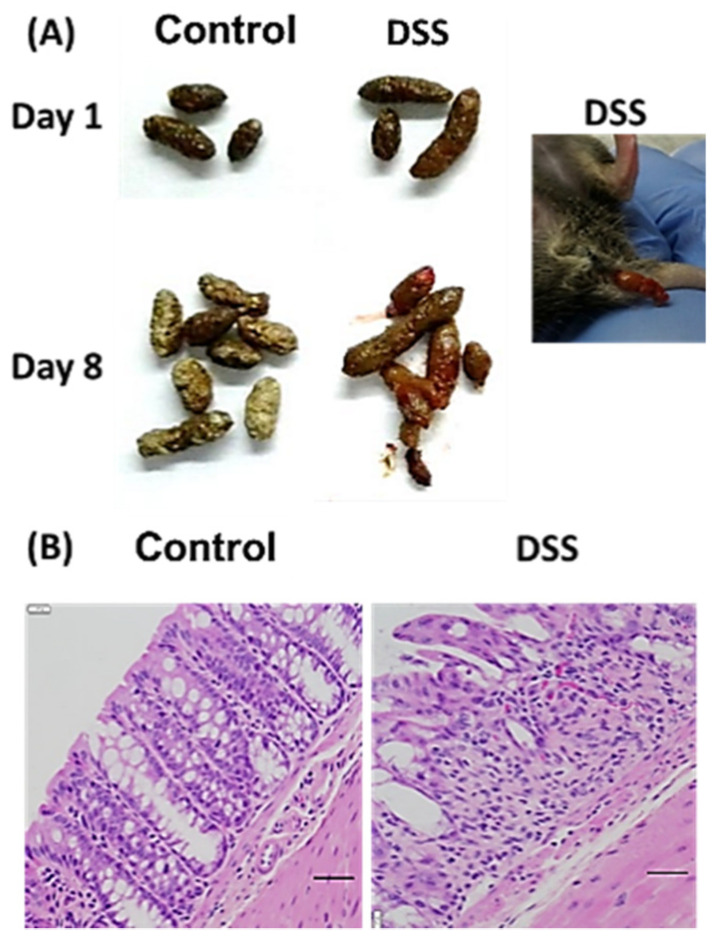
(**A**) Feces and (**B**) colon histology of mice with or without DSS treatment. DSS-treated mice have apparent inflammatory cell infiltration. H&E-stained colon sections (magnification, ×100).

**Figure 2:**
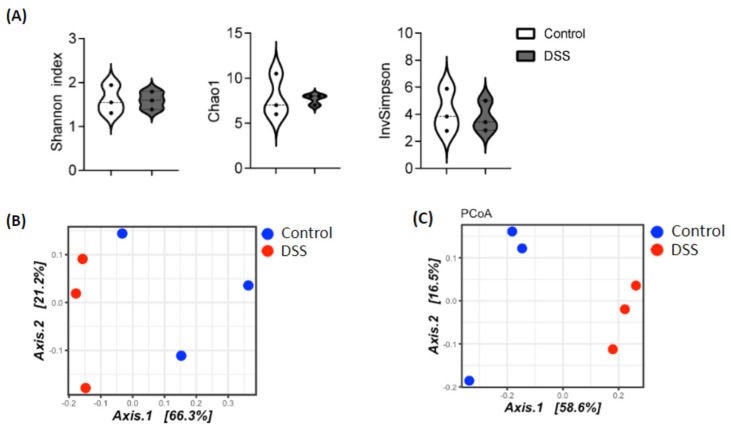
(**A**) Alpha diversity index from 16S rRNA sequencing, (**B**) Principal coordinates analysis (PCoA) plot of the Bray-Curtis dissimilarities of cecal microbiota 16S rRNA profiling Control and DSS treated mice (n = 3/group) at the family (**C**) and genus level.

Phenotypic changes were noted in response to DSS intake. Colitis mice had loose and blood-stained feces (Figure 1A). Additionally, colitis mice had tissue damage, including mucosal erosion, infiltration of inflammatory cells, and crypt distortion and disappearance (Figure 1B). Based on the scoring criteria detailed in Table 1 and Table 2, the healthy mice had a score of zero, while the colitis mice had an average of 28. Diversity plots as well as a Bray–Curtis dissimilarity were also conducted, with the Bray–Curtis dissimilarity quantifying the compositional dissimilarity between two samples or groups at the family and genus level (Figure 2).

### 3.2. Changes in Inflammatory Cytokines

**Figure 3:**
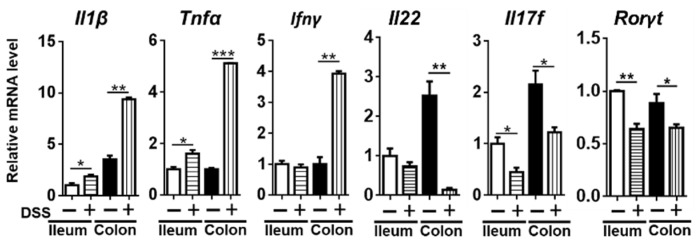
The expression of inflammatory genes in the ileum and colon of mice with or without DSS treatment. Data are expressed as mean ± SD. n = 9 per group. Student’s *t*-test. * *p* < 0.05, ** *p* < 0.01, *** *p* < 0.001.

Compromised immunity likely leads to increased inflammatory signaling. Thus, we studied the expression of pro- and anti-inflammatory genes. In colitis mice, the mRNA levels of *Il-1β* (interleukin 1 beta), *Ifn-γ* (interferon gamma), and *Tnf-α* (tumor necrosis factor alpha) had increased 3–5 folds in the colon. In contrast, only the mRNA levels of *Il-1β* and *Tnf-α* had a modest increase in the ileum. Additionally, the expression of *Il-22* (interleukin 22), *Il-17f* (interleukin 17f), and *Rorγt* (RAR-related orphan receptor gamma) were reduced in colitis mice (Figure 3). *IL-22* as a member of the *IL-10* family can prevent tissue damage and aid in repair. In total, the colon had more changes in inflammatory signaling than the ileum.

### 3.3. Dysbiosis Occurs in Colitis Mice

**Figure 4:**
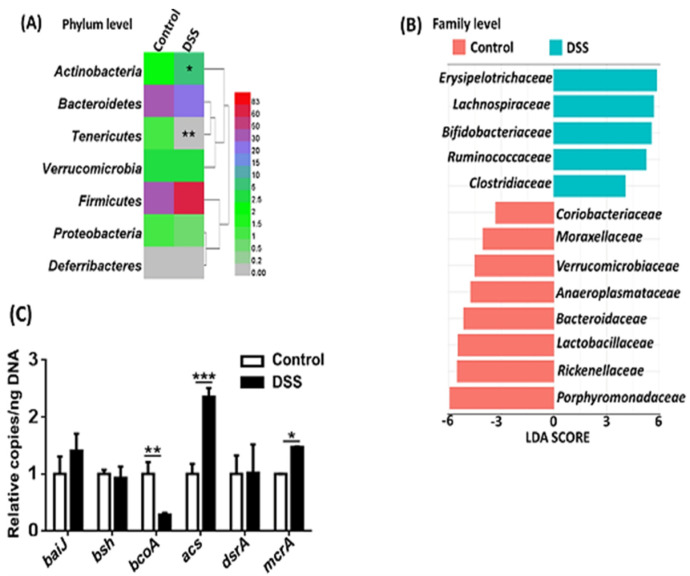
Alteration of gut microbiota composition in DSS-induced colitis mice. (**A**) Change in bacteria abundance at the phylum level. Data are expressed as mean ± SD. n = 9 per group. Mann-Whitney U test, * *p* < 0.05, ** *p* < 0.01. (**B**) Difference in family level identified by LEfSe (LDA > 2, *p* < 0.05). (**C**) Quantification of bacteria genes that have known functions. Copy number per ng DNA was calculated in control and DSS-treated mice. Data are expressed as mean ± SD. n = 4 per group. Two-way ANOVA with Bonferroni correction. * *p* < 0.05, ** *p* < 0.01, *** *p* < 0.001.

Gut microbiota plays a pivotal role in the intestinal barrier and immune function. We studied the gut microbiota profile in healthy and colitis mice. At the phylum level, *Actinobacteria* were increased while *Tenericutes* were markedly decreased in colitis mice (Figure 4A). There were 13 differentially abundant families with linear discriminant analysis (LDA) scores higher than 2.0 as shown by linear discriminant analysis effect size (LEfSe) (Figure 4B). The abundance of *Erysipelotrichaceae*, *Lachnospiraceae*, *Bifidobacteriaceae*, *Ruminococcaceae*, and *Clostridiaceae* were increased, whereas *Porphyromonadaceae*, *Rickenellaceae*, *Lactobacillaceae*, *Bacteroidaceae*, *Anaeroplasmataceae*, *Verrucomicrobiaceae*, *Moraxellaceae*, and *Coriobacteriaceae* were reduced in colitis mice.

Additionally, the abundance of cecal bacterial genes with known functions was quantified. The abundance of butyrate-producing *bcoA* (butyryl-CoA: acetate CoA transferase) was reduced, while acetate-generating *acs* (Acetyl-CoA Synthase) as well as methane producer *mcrA* (5-methylcytosine-specific restriction enzyme A) were increased in colitis mice (Figure 4C). Other studied genes, including *baiJ* (bile acid-inducible operon J) and *bsh* (bile salt hydrolase), as well as sulfite-producing *dsrA* (dissimilatory sulfite reductase A), remained unchanged due to the development of colitis.

### 3.4. The Expression of Genes Implicated in RA Signaling

To examine the impact of colitis on RA signaling, we studied the expression of *Aldh1a1* (aldehyde dehydrogenase 1 family member A1), *Cyp26a1* (cytochrome P450 family 26 subfamily A member 1), *Cyp26b1* (cytochrome P450 family 26 subfamily B member 1), RA transport *Rbp4* (retinol binding protein 4), *Crabp1* (cellular retinoic acid binding protein 1), *Crbp1* (cellular retinol binding protein 1), and RA receptor *Rarβ* (retinoic acid receptor beta).

**Figure 5:**
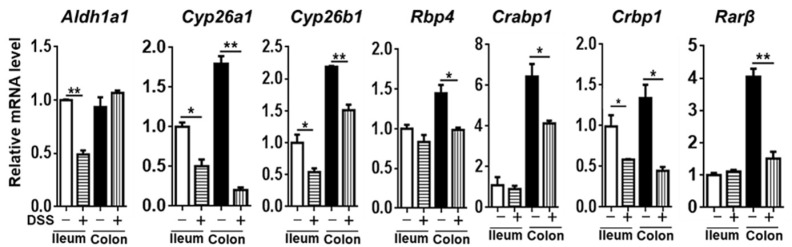
RA signaling gene expression in the ileum and colon of mice treated with or without DSS. Data are expressed as mean ± SD. n = 9 per group. Student’s *t*-test. * *p* < 0.05, ** *p* < 0.01.

In the intestine, many of the studied genes had reduced expression levels, as shown in Figure 5. Reduced *Aldh1a1* was found in the ileum, but not in the colon. The expression of *Cyp26a1* and *Cyp26b1* was found in both locations. Importantly, reduced *Rarβ* was only found in the colon, suggesting that colitis had a greater impact on RA signaling in the colon than in the ileum (Figure 5).

**Figure 6:**
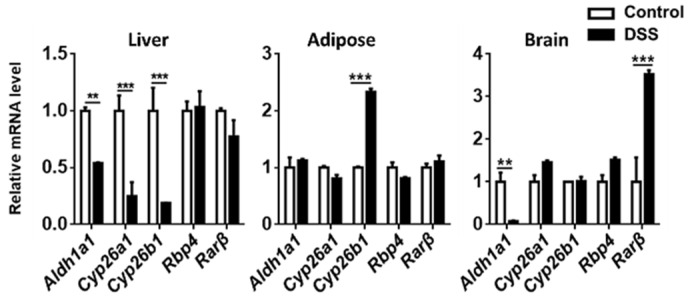
RA signaling gene expression in the liver, adipose, and brain of mice treated with or without DSS. Data are expressed as mean ± SD. n = 9 per group. Student’s *t*-test. ** *p* < 0.01, *** *p* < 0.001.

In the liver, colitis reduced the mRNA levels of *Aldh1a1, Cyp26a1,* and *Cyp26b1,* suggesting reduced production as well as oxidation (Figure 5). In adipose tissue, colitis mice had increased *Cyp26b1* mRNA levels without increasing the expression of *Aldh1a1,* suggesting reduced RA concentration. In the brain, the colitis mice had reduced *Aldh1a1* but increased *Rarβ* mRNA, which might suggest reduced production and perhaps a compensatory induction of the receptor (Figure 6). Together, there was a systemic impact on RA signaling in response to colitis.

### 3.5. The Expression of RA-Regulated Gut-Homing Genes

**Figure 7:**
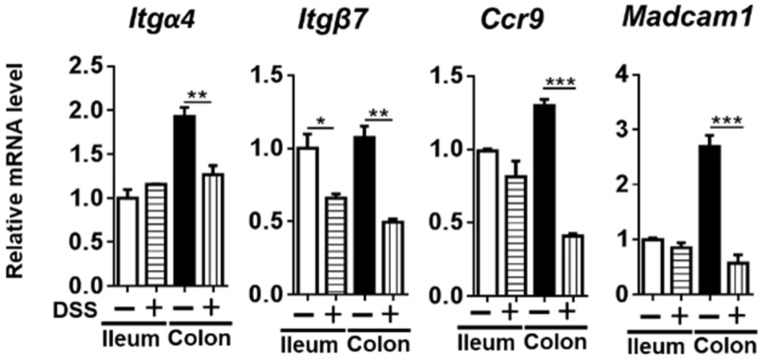
Gut-homing signaling gene expression in the ileum and colon of mice treated with or without DSS. Data are expressed as mean ± SD. n = 9 per group. Student’s *t*-test. * *p* < 0.05, ** *p* < 0.01, *** *p* < 0.001.

As mentioned above, RA is an immunomodulator that induces gut-homing molecules α4β7 integrin and CCR9. α4β7 integrin binds to endothelial MAdCAM-1 (mucosal vascular addressin cell adhesion molecule 1), which also can be induced in response to RA. In the colon of colitis mice, the mRNA levels of *Itgα4* (integrin subunit alpha 4), *Itgβ7* (integrin subunit beta 7), and *Ccr9* (C-C motif chemokine receptor 9) were all reduced compared with the healthy controls. In addition, the level of *Madcam1* mRNA was also reduced in the colon of colitis mice (Figure 7). Overall, reduced RA signaling was accompanied by decreased gut-homing genes in the colon of colitis mice.

## 4. Discussion

RA is essential for differentiation and maintenance of epithelial integrity [33,34]. The current study supports the scenario that reduced RA signaling is implicated in the development of IBD [35,36]. Consistently, obesity, as a risk for colitis, compromises RA signaling. It is alarming that the prevalence of obesity is rising in parallel with the incidence of IBD [12,37]. Hence, early detection of reduced RA signaling can be useful in prevention.

In the current study, because inflammation was induced by a chemical rather than a diet, it stresses the significance of inflammation in dampening RA signaling. When it comes to nutrition, serum vitamin A concentration is used as a standard test. However, vitamin A is not biologically active and vitamin A deficiency is rarely seen in Western countries. It would be critical to study RA signaling in tissues rather than detecting vitamin A deficiency in serum.

Our data support that inflammation and reduced RA signaling go together found in the liver, brain, and adipose tissues. Based on the known functions of RA in each organ, it is likely that colitis not only affects gut immunity but also impacts hepatic detoxification, regeneration, lipid metabolism, adipogenesis, and even neuroplasticity.

Gut homing plays a pivotal role in the development of IBD. Overexpression of integrin α4β7 exacerbates IBD [37], and anti-α4β7 antibody vedolizumab can suppress the recruitment of inflammatory T and B cells into the gut, thus it is used for IBD treatment [38,39]. In contrast, the absence or deletion of α4β7 deteriorates colitis [40]. Hence, it is essential to maintain the homeostasis of α4β7 signaling. As our data showed, DSS-reduced RA signaling was accompanied by decreased expression of gut-homing genes that are regulated by RA. These changes likely compromise lymphocyte homing to the gut and impair host defense.

Disequilibrium between pro-inflammatory and anti-inflammatory cytokines plays a crucial role in the pathogenesis of IBD. The imbalance results in disease perpetuation and tissue destruction [41]. The mRNA level of *Il-22*, an important anti-inflammatory cytokine, was decreased in colitis mice. IL-22 plays a beneficial role in IBD by inducing antimicrobial molecules as well as proliferative and anti-apoptotic pathways to stimulate tissue repair [42,43]. It has been shown that RA induces IL-22 production in γδ T cells [44]. Thus, impairment of RA signaling likely contributes to reduced *Il-22* mRNA levels in colitis mice.

IL-17F plays a key regulatory role in host defense and inflammatory diseases [45,46]. IL-17F prevents pathogen invasion at epithelial and mucosal barriers by inducing effector cells to secrete pro-inflammatory cytokines, anti-pathogenic peptides, chemokines, and mucin [47,48]. However, IL-17F also promotes the inflammatory pathology of colitis [49,50]. Reduced expression of *Il-17f* mRNA in colitis mice might compromise the intestinal defense mechanism. Consistently, the gene encoding the transcriptional factor RORγ for IL-17 expression was also reduced in colitis animals. Moreover, the expression of *Tnf-α*, *Il-1β*, and *Ifn-γ* was increased. Overall, an increase in inflammatory cytokines and a reduction in anti-inflammatory cytokines were found in colitis mice, and this disequilibrium likely contributes to the development of colitis.

Dysbiosis is implicated in the pathogenesis of IBD, and fecal microbiota transplantation has been used in IBD treatment [51,52]. However, the interplay and regulation between gut flora and the host immune system has not been fully established [53,54]. Our data showed that *Erysipelotrichaceae* was increased after DSS treatment. *Erysipelotrichaceae* has been found to be enriched in colorectal cancer [55,56]. In addition, the expansion of *Erysipelotrichaceae* in mice with chronic ileal inflammation has also been observed [57]. Furthermore, it has been shown that the abundance of *Erysipelotrichi* is positively correlated with TNFα levels [58].

*Lachnospiraceae* also increased in colitis mice. In contrast, *Lactobacillaceae*, which includes *Lactobacilli*, an important probiotic, decreased in colitis mice. Interestingly, an increase in *Lachnospiraceae,* as well as a reduction in *Lactobacillaceae,* has been shown in murine lupus models. Moreover, RA can improve lupus symptoms via restoration of intestinal *Lactobacillaceae* colonization [59]. Moreover, *Clostridiaceae* was expanded in colitis mice. It has been shown that IBD patients are susceptible to infection of *Clostridium difficile*, which belongs to the *Clostridiaceae* family [60]. Moreover, IBD patients with *C. difficile* infections have severe flares and high rates of colectomy [61].

*Verrucomicrobiaceae* was also reduced in colitis mice. Our previous study shows that epigallocatechin-3-gallate-regulated metabolic health benefits are accompanied by the expansion of *Verrucomicrobiaceae* [62]. In addition, *A. muciniphila* supplementation can reverse fat-mass gain, metabolic endotoxemia, adipose tissue macrophage infiltration, and insulin resistance caused by a high-fat diet [63].

As a bacterial metabolite, butyrate, which can stimulate RA production, plays an important role in maintaining gastrointestinal health [64]. *BcoA* is a key gene for butyrate synthesis [65]. Our data showed that butyrate-producing bacteria were reduced in colitis mice, suggesting the reduced RA signaling might be in part due to a decrease in butyrate-generating bacteria. Butyrate is also an energy source for colonocytes. Thus, the reduction in *bcoA* likely affects mucosal tissue renewal and integrity [66].

Acetate accounts for the highest percentage of short-chain fatty acids, but it does not have histone deacetylase inhibitory properties. Acetate is produced by most enteric bacteria such as *Lactobacillus* spp., which are reduced in colitis mice. Butyrate-producing bacteria utilize acetate [67]. An increased copy number of *acs*, the acetate synthesis gene, was found in colitis mice suggesting that acetate accumulation and gut microbes are imbalanced. Moreover, increased *mcrA* (methyl coenzyme M reductase), which generates methane, has been found to be associated with the development of IBD, colon cancer, and obesity [68,69]. Consistently, the copy number of *mcrA* was increased in colitis mice. Together, colitis-associated dysbiosis has a functional consequence.

**Figure 8:**
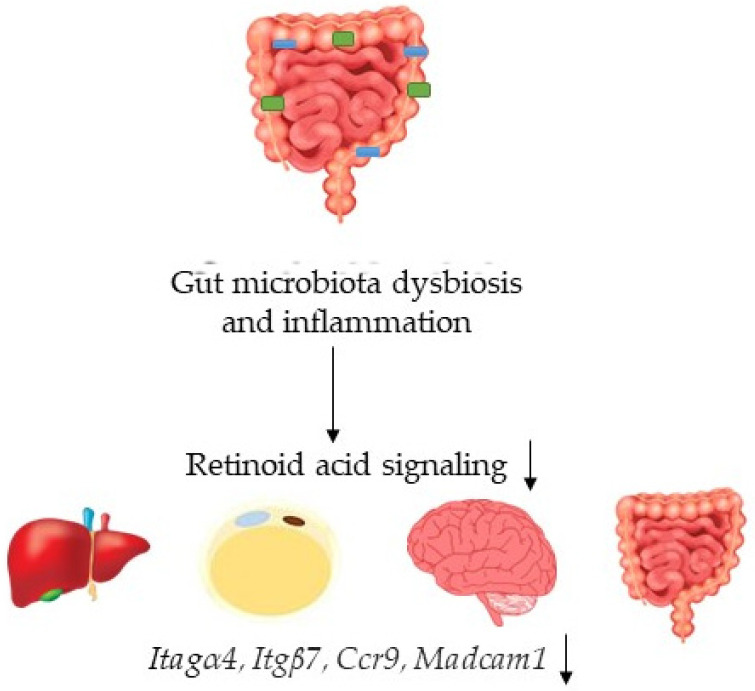
DSS-induced gut dysbiosis and colitis cause compromised retinoic acid signaling at the systemic level. Downward arrows next to signaling or gene names indicate inhibition.

In conclusion, dysbiosis associated colon inflammation has a negative impact on RA signaling in other organs (Figure 8). Additionally, the data suggest a potential for probiotics to be useful to improve RA signaling at the systemic level.

## Figures and Tables

**Table 1:** Scoring criteria for macroscopic observation of colon and ileum.

Morphology of the Colon and Ileum	Score
No ulcers, no inflammation	0
No ulcers, local mucosal congestion and edema	1
Single ulceration, but no hyperemia of mucosa	2
Single ulceration, mucosal inflammation	3
Multiple ulcers and mucosal inflammation	4
Ulcer area >2 cm, severe erosion, mucosal hyperemia and edema	5

**Table 2:** Histopathological scoring criteria of ileum and colon.

Histopathological Manifestations of the Ileum and Colon	Score
No obvious inflammation reaction	0
Low-level inflammatory cell infiltration, no damage to intestinal villi structure	1
Moderate inflammatory cell infiltration, deepening of the intestinal crypts, thickening of the intestinal wall but not invading the muscle layer	2
High levels of inflammatory cell infiltration, vascular proliferation, thickening of the intestinal wall and invasion of the muscle layer	3
Infiltration of inflammatory cells, reduction in goblet cells, vascular proliferation, thickening of the intestinal wall and invasion of the muscle layer	4

**Table 3:** Primers used for qPCR.

Gene	Primers (5′–3′)	Tm	Amplicon Size	NCBI Accession Number
*Aldh1a1*	F:AGGCCCTCAGATTGACAAGGAACA	63.89	135	NM_013467
	R:AACACTGTGGGCTGCACAAAGAAG	64.25		
*Ccl25*	F:CCAAGGTGCCTTTGAAGACT	58.01	400	NM_009138
	R:TCCTCCAGCTGGTGGTTACT	60.18		
*Ccr9*	F:CTTGCCACTCTTCCCTTCTG	58.18	170	NM_001166625
	R:GCCTTCATGGCCTGTACAAT	58.23		
*Crabp1*	F:GCTGGCCAACGATGAGCTAA	60.74	74	NM_013496
	R:ACTCCCGGACATAAATTCT GTG	58.74		
*Crbp1*	F:AGCAGGTGAGAAGGGATAAAG GT	61.08	75	NM_011254
	R:CGTGTTCCGTGGCTTCTGAT	60.67		
*Cyp26a1*	F:GCACAAGCAGCGAAGAAGGTGAT	64.38	104	NM_007811
	R:ACTGCTCCAGACAACTGCTGACTT	64.31		
*Cyp26b1*	F:GGCGGCTACCGCACTGT	62.54	88	NM_175475
	R:TGTCTCGGATGCTAT CATGACACT	61.44		
*Il1β*	F:AAGATGAAGGGCTGCTTCCA	59.30	78	NM_008361
	R:GTGCTGCTGCGAGATTTGAA	59.48		
*Il-17a*	F:TCCAGAAGGCCCTCAGACTA	59.29	248	NM_010552
	R:ACACCCACCAGCATCTTCTC	59.67		
*Il22*	F:TTGAGGTGTCCAACTTCCAGCA	62.08	97	NM_016971
	R:AGCCGGACGTCTGTGTTGTTA	61.96		
*Il-6*	F:GTTGCCTTCTTGGGACTGATG	59.18	90	NM_001314054
	R:GGGAGTGGTATCCTCTGTGAAGTCT	63.10		
*Infγ*	F:AGCTCTTCCTCATGGCTGTT	59.01	148	NM_008337
	R:TCCTTTTGCCAGTTCCTCCA	59.15		
*Itgα4*	F:GCCTGGAGGAGAGGGATAAC	58.95	159	NM_010576
	R:CAGAAGGCATGACGTAGCAA	58.27		
*Itgβ7*	F:CTACGACTCTGGGCTCTTGG	59.54	193	NM_013566
	R:ACAGGTCAGCCTCAGAGCAT	60.91		
*Madcam1*	F:GCATGGTGACCTGGCAGT GAAG	63.96	397	NM_001358785
	R:GGCAGCAGTATCCTCTCTGTAC	59.96		
*Rarβ*	F:GCACTGACGCCATAGTGGTA	59.83	88	NM_001289762
	R:CACCATCTCCACTTCCTCCT	58.42		
*Rbp4*	F:TCTGTGGACGAGAAGGGTCAT	60.55	72	NM_001159487
	R:CACTTCCCAGTGCTCAGAAGA	60.22		
*Rorγt*	F:ACCTCCATGCCAGCTGTGTGCTGTC	70.66	725	NM_011281
	R:CAAGTTCAGGATGCCTGGTTTCCTC	63.79		
*Tnfα*	F:TCGAGTGACAAGCCTGTAG	56.51	121	NM_013693
	R:GTTGGTTGTCTTTGAGATCC	54.22		

*Aldh1a1:* Aldehyde Dehydrogenase 1 Family Member A1; *Ccl25:* C-C Motif Chemokine Ligand 25; *Ccr9:* C-C Motif Chemokine Receptor 9; *Crabp1:* Cellular Retinoic Acid Binding Protein 1; *Crbp1:* Cellular Retinol Binding Protein Gene 1; *Cyp26a1:* Cytochrome P450 Family 26 Subfamily A Member 1; *Cyp26b1:* Cytochrome P450 Family 26 Subfamily B Member 1; *Il1β:* Interleukin 1 Beta; *Il-17a:* Interleukin 17A; *Il22:* Interleukin 22; *Il-6:* Interleukin 6; *Itg4α:* Integrin Subunit Alpha 4; *Itgβ7:* integrin subunit beta 7; *Madcam1:* Mucosal Vascular Addressin Cell Adhesion Molecule 1; *Rarβ:* Retinoic Acid Receptor Beta; *Rbp4:* Retinol Binding Protein 4; *Rorγt:* Retinoic acid receptor-related-orphan-receptor-gamma t; *Tnfα:* Tumor Necrosis Factor-Alpha.

**Table 4:** Primers used to quantify bacteria gene copy numbers.

Gene	Forward (5′–3′)	Reverse (5′–3′)
*acs*	CTYTGYCAGTCMTTYGCBCC	CCCATAAABCCYGGDGTYTG
*baij*	TCAGGACGTGGAGGCGATCCA	TACRTGATACTGGTAGCTCCA
*bcoa*	GCIGAICATTTCACITGGAAYWSITGGCAYATG	CCTGCCTTTGCAATRTCIACRAANGC
*bsh*	ATGGGCGGACTAGGATTACC	TGCCACTCTCTGTCTGCATC
*dsra*	GCCGTTACTGTGACCAGCC	GGTGGAGCCGTGCATGTT
*mcra*	TTCGGTGGATCDCARAGRGC	GBARGTCGWAWCCGTAGAATCC

*acs*: Acetyl-CoA Synthase; *baij*: bile acid inducible operon J; *bcoa*: butyryl-CoA; *bsh*: bile salt hydrolase; *dsra*: dissimilatory sulfite reductase A; *mcra*: 5-methylcytosine-specific restriction enzyme A.

## Data Availability

The data in this study are available upon request from the corresponding author.
